# Bone scintigraphy outperformed anatomic images in frostbite injuries: a case report

**DOI:** 10.1186/s41824-022-00146-1

**Published:** 2022-11-10

**Authors:** Brunela Ronchi, Gustavo Agustin Peña, Albertti Carla

**Affiliations:** Department of Nuclear Medicine, Foundation School of Nuclear Medicine (FUESMEN), Mendoza, Argentina

## Abstract

Frostbite is a localized cold thermal injury, as a result of tissue exposure to temperatures below freezing point for a prolonged period of time. The spectrum of injury is broad; thus, frostbite injuries may have deleterious effects with the possibility of losing part or whole extremities. We aim to present the case of a 38-year-old male patient evaluated with multiphase technetium-99m-methylene diphosphonate bone scintigraphy. This methodology may accelerate clinical management of frostbite injuries because it provides precise clinical-imaging correlation by determining the extent of injury and can accurately predict the level of amputation if required.

## Introduction

Frostbite is a localized cold thermal injury, caused by tissue exposure to temperatures below freezing point for a prolonged period of time (Handford et al. 2014). Treatment of frostbite injuries is a complex and multimodal process depending on the stage and grade of injury (Manganaro et al. 2019; Millet et al. 2016). The main focus of therapy is to prevent further tissue injury and to rewarm the tissue as soon as possible (Manganaro et al. 2019; Millet et al. 2016). Consequently, bone scans are useful not only to predict tissue activity, but also to assess the response of the lesion to treatment with greater precision.

## Case presentation

**Fig. 1:**
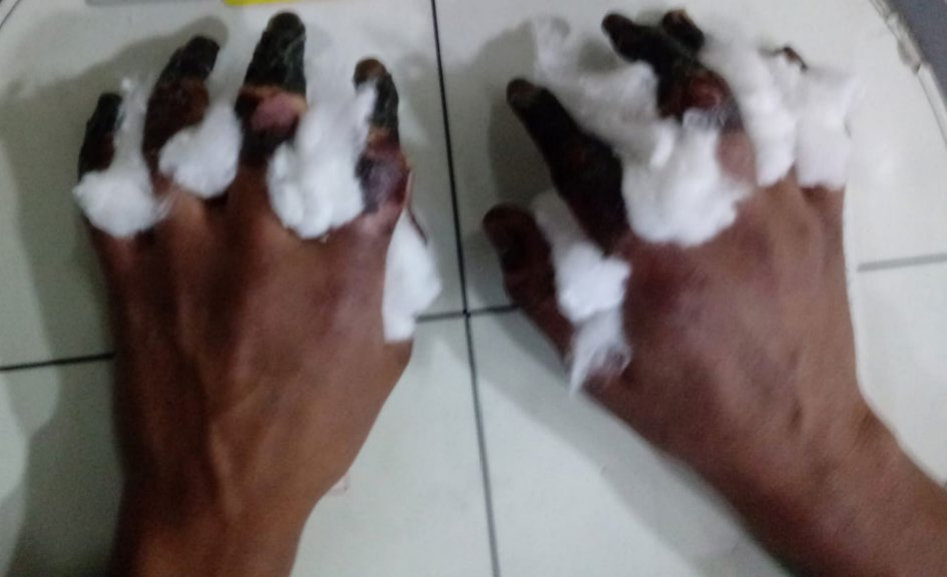
Physical examination demonstrated gangrene involving all digits of both hands, concluding a fourth-degree frostbite. Therefore, multiphase 99mTc-MDP bone scintigraphic imaging was performed to both hands

**Fig. 2:**
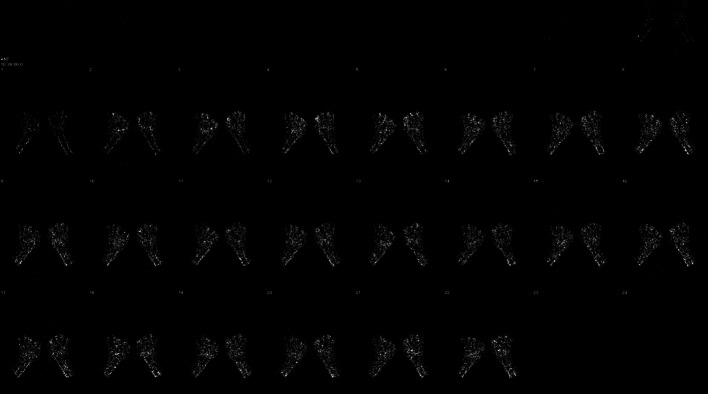
Dynamic multiphase 99mTc-MDP bone scintigraphic. Images of the hands show absent tracer uptake throughout the bilateral phalanges in the palmar blood flow phase immediately after the radiotracer injection

**Fig. 3:**
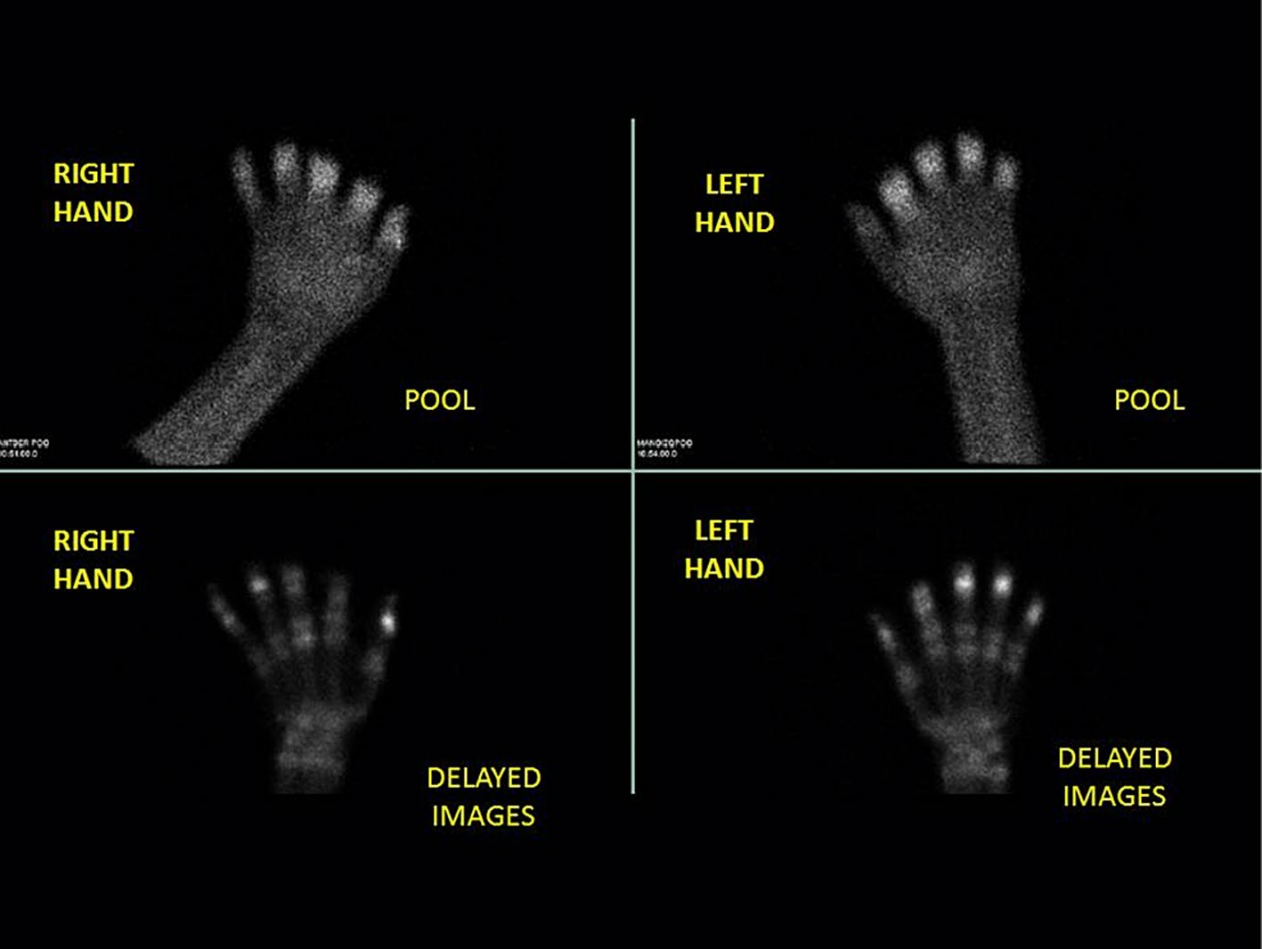
Multiphase 99mTc-MDP bone scintigraphic. Multiphase 99mTc-MDP bone scintigraphic images of the hands show palmar soft tissue phase (above) and 4-h delayed phase (below)

We present a healthy 38-year-old male mountain climber who intended to reach the peak of the tallest mountain in the region named Aconcagua which is 6962 m high. Due to an unexpected snow storm, he got caught in a precarious shelter without the necessary equipment to spend the night. Thus, the next day he was rescued by national guards with frostbite injuries in both hands (Fig. 1). Multiphase 99mTc-MDP bone scintigraphy was performed afterward (Figs. 2, 3).

Unfortunately, despite the thrombolytic, thermal and antibiotic treatment established once the patient arrived at the emergency department, the amputation of the last phalanges was necessary based on the result of the bone scintigraphy.

## Discussion

Frostbite is a localized cold thermal injury, as a result of tissue exposure to temperatures below freezing point (typically − 0.55 °C, but can occur as high as 2 °C) for a prolonged period of time (Handford et al. 2014; Manganaro et al. 2019). Typically, it involves young men between 30 and 50 years old firstly cited in the military and in countries with extreme temperatures. Later in rural areas, it has been described among homeless, malnutrition, smoking, alcoholics, substance abusers, psychiatric patients, and recently in those individuals who practice winter sports (Handford et al. 2014; Manganaro et al. 2019; Millet et al. 2016). Systemic diseases such as peripheral vascular disease like the Raynaud phenomenon, vasculitis, diabetes mellitus, heart failure are predisposed factors for individuals to be affected by frostbite (Millet et al. 2016; Ingram and Raymond 2013). Cold injuries affect extremities in the greatest proportion: hands and feet predominantly with ears, cheek, nose and genitalia reported in a fewer rate (Manganaro et al. 2019; Millet et al. 2016). The severity of frostbite injury depends on environmental temperature, the wind chill factor and the length of exposure being the duration of exposure the utmost important factor (Manganaro et al. 2019; Imray et al. 1007).

The spectrum of injury is wide, and thus, frostbite injuries may have devastating effects with the possibility of losing part or whole extremities. Therefore, it is paramount for medical staff to have the proper knowledge of the benefits of imaging techniques to provide a certain diagnostic approach. Magnetic resonance and bone scintigraphy are superior to X-rays and allow early intervention in cases of severe frostbite, thus preventing secondary infection (Cauchy et al. 2000; Barker et al. 1997).

**Fig. 4:**
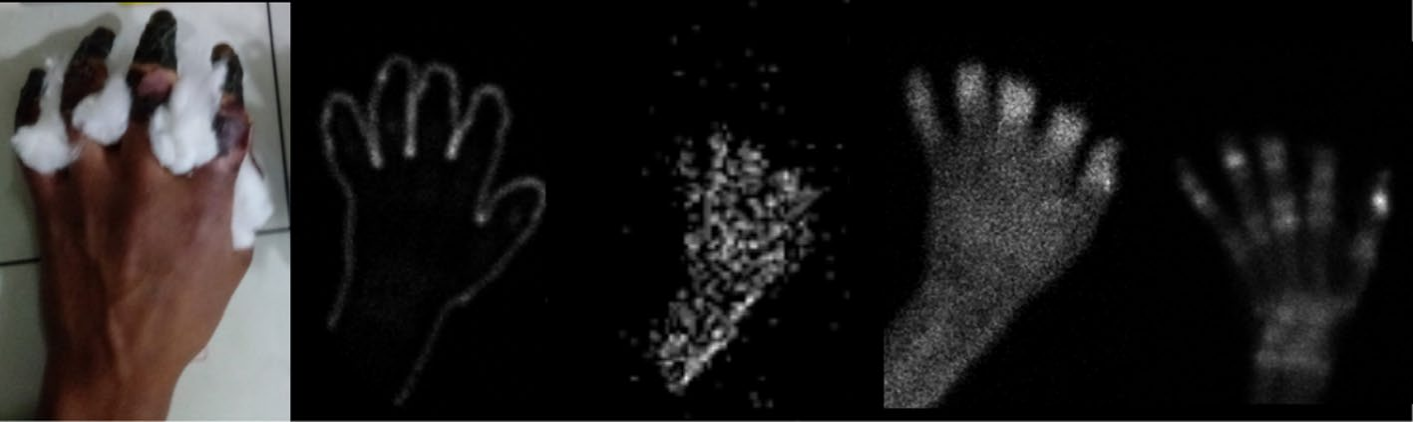
High clinical-imaging correlation showed for the right hand. Clinical appearance of a fourth-grade frostbite injury. Peripheral marking of the hand with 99mTc-MDP to delimit the contour. Multiphase 99mTc-MDP bone scintigraphic images. Note the increased tracer uptake between the phalangeal joints related to the level of necrosis on the delayed phase image

Multiphase technetium-99m-methylenediphosphonate (99mTc-MDP) bone scintigraphy may hasten clinical management of frostbite injuries as it furnish high and precise clinical-imaging correlation by determining the extent of injury and can accurately predict the level of amputation if required (Fig. 4) (Manganaro et al. 2019; Cauchy et al. 2000).

### Pathogenesis

Basically, frostbite injuries occur due to the formation of ice crystals intracellularly and extracellularly which provokes microcirculatory dysfunction, osmotic gradient alterations and inflammatory reactions (Manganaro et al. 2019; Gao et al. 2021). Intracellular ice crystal formation stimulates a cascade of deleterious effects resulting in the production of prostaglandins, cytokines and oxygen free radicals as an intent to remove ice crystals and preserve cells. Notwithstanding, these products impoverish the cell environment and produce detrimental effects, such as hypoxia and microthrombosis, creating a vicious circle where the production of more substances is stimulated, resulting in cell death (Manganaro et al. 2019; Murphy et al. 2000).

### Imaging

#### Radiology

Radiography is commonly the first diagnostic technique available, and it may be used to examine the underlying bone, however lacking pivotal information as it is the vasculature examination. X-rays may identify radiopaque foreign bodies or occult fractures (Millet et al. 2016). Radiographic findings include nonspecific changes. In an initial stage, soft tissue changes as oedema or marked soft tissue swelling with tissue distortion (Millet et al. 2016). In late stages, radiography may show osteopenia and osteoporosis (Millet et al. 2016; Gao et al. 2021).

#### Magnetic resonance (MR) angiography

MR may be used in the initial evaluation of frostbite injuries. On T1- and T2-weighted sequences, they show increased signal intensity in all muscle groups in initial stages and signal absent in later stages. MR angiography is a noninvasive tool to assess vasculature of occluded vessels and has a higher soft tissue resolution. MR angiography may have limited clinical utility in helping to define persistently occluded vessels and demarcate the soft tissue ischemic border in patients who present more than 24 h after injury (Millet et al. 2016; Barker et al. 1997).

#### Bone scintigraphy

Multiphase technetium-99m-methylenediphosphonate (99mTc-MDP) bone scintigraphy assesses tissue viability in an effort to allow early debridement of soft tissue and early coverage of ischemic bony structures with an almost unerring accuracy: specificity 99% and sensitivity 96% (Imray et al. 1007; Cauchy et al. 2000).

Bone scintigraphy is commonly used with bisphosphonates labeled with 99mTc, which is a compound that binds to hydroxyapatite crystals present in the surface of viable bone. Another key point is that uptake depends on vascularization and the activity of new bone formation or blast activity. Consequently, non-viable areas of bone due to freezing injuries are visualized as photopenic or cold areas with little or no vascularization in early blood flow and pool images.

Bone scintigraphy is a noninvasive technique with low radiation exposure and easy access. Thus, it is the elective diagnosis procedure particularly given that contraindications and side effects are practically nonexistent or relative (pregnancy).

Ideally, an initial bone scan is appropriate within the day 2 to 4 after exposure albeit, the transfer of the patient from the site of exposure may be delayed particularly in mountaineers. A second bone scan should be performed between the day 7 to 10 to follow the evolution of areas of low tracer uptake (Millet et al. 2016; Cauchy et al. 2000).

Imaging protocol includes a three-phase scan. Firstly, an early tissue phase or planar blood flow phase immediately after intravenous administration of 20–30 mCi 99mTc-MDP is useful to identify the vasculature and to delimit the level of vascularization (Fig. 2). Secondly, planar soft tissue phase images (known as blood pool phase images) are acquired in 3–5-min intervals for up to 10 min after tracer injection which allows the evaluation of soft tissues (Fig. 3). Lastly, bone phase is taken approximately between 2 and 4 h later which enables bone perfusion and viability. Particularly in our center, we have decided to extend the last phase up to 4–6 hs to properly assess bone viability (Fig. 3).

Bone scans can be used not only to predict tissue activity, but also to assess the response of the lesion to treatment. Reduced perfusion (not absent) indicates the possibility that viable tissue exists and needs specific therapy such as thrombolytic treatment (Gao et al. 2021). On the contrary, the possibility of amputation is high when the tissue uptakes are absent (photopenic or cold areas) (Millet et al. 2016; Gao et al. 2021).

## Conclusions

Frostbite injuries may be varied and its clinical spectrum. However, it is complex to precisely identify viable tissue with only clinical examination. Hence, a variety of imaging diagnostic techniques have intended to address this rising issue with limited results. Bone scintigraphy provides a strong clinical-imaging correlation that identifies the depth and extent of tissue injury and can reliably predict the level of amputation if necessary.

## Data Availability

The data supporting the conclusions of this article is included within the article.
